# Genome-Wide Identification and Evolutionary Analysis of the bHLH Transcription Factor Family in *Rosa roxburghii*

**DOI:** 10.3390/ijms27020912

**Published:** 2026-01-16

**Authors:** Yuan-Yuan Li, Li-Zhen Ling, Shu-Dong Zhang

**Affiliations:** 1 College of Life and Health, Dalian University, Dalian 116622, China; li_yuanyuan9@foxmail.com; 2 Key Laboratory for Specialty Agricultural Germplasm Resources Development and Utilization of Guizhou Province, Liupanshui Normal University, Liupanshui 553004, China; primula_ling@foxmail.com

**Keywords:** bHLH family, *Rosa roxburghii*, gene duplication, functional divergence

## Abstract

The basic/helix-loop-helix (bHLH) transcription factors are crucial regulators of plant development and stress responses. In this study, we conducted a genome-wide analysis of the bHLH family in *Rosa roxburghii*, an economically important fruit crop. A total of 89 non-redundant *RrbHLHs* were identified and unevenly distributed across the seven chromosomes. Phylogenetic analysis classified them into 23 subfamilies and 7 *Arabidopsis* subfamilies were absent, indicating lineage-specific evolutionary trajectories. Conserved motif and gene structure analyses showed that members within the same subfamily generally shared similar architectures, yet subfamily-specific variations were evident, suggesting potential functional diversification. Notably, key residues involved in DNA-binding and dimerization were highly conserved within the bHLH domain. Promoter analysis identified multiple *cis*-acting elements related to hormone response, stress adaptation, and tissue-specific regulation, hinting at broad regulatory roles. Expression profiling across fruit developmental stages and in response to GA_3_ treatment revealed dynamic expression patterns. Furthermore, 21 duplicated gene pairs (17 segmental and 4 tandem duplicated pairs) were identified, with most evolving under purifying selection. Detailed analysis of these pairs revealed that segmental duplication, coupled with structural variations such as exon indels, dissolution/joining, and exonization/pseudoexonization, substantially contributed to their functional divergence during evolution. Our results provide a basis for understanding the evolution and potential functions of the *RrbHLHs*.

## 1. Introduction

The basic/helix-loop-helix (bHLH) transcription factors represent a substantial and evolutionarily conserved superfamily of regulatory proteins in eukaryotes [1,2]. A typical bHLH domain encompasses approximately 60 amino acids and comprises two functionally distinct regions: an N-terminal basic region that recognizes and binds to specific DNA motifs, such as the E-box (CANNTG) in target gene promoters, and a C-terminal helix-loop-helix (HLH) region that facilitates protein dimerization [3,4,5]. As the second-largest transcription factor family in plants, *bHLHs* are crucial in various biological processes, including growth and development, secondary metabolism, hormone signaling, and responses to biotic and abiotic stresses [1,6]. For instance, bHLH members in *Arabidopsis thaliana* such as *FIT* and *PYE* are key regulators of iron homeostasis, while *MYC2* and *TT8* are involved in jasmonic acid signaling and anthocyanin biosynthesis, respectively [7].

The functional diversity of the bHLH family is intricately linked to its structural complexity and evolutionary history, particularly through gene duplication events. Whole-genome duplication (WGD), segmental duplication, and tandem duplication have been identified as significant drivers of the expansion of the bHLH family across plant species [8]. For example, studies on pummelo (*Citrus grandis*) [9] and wintersweet (*Chimonanthus praecox*) [10] have revealed that 47 and 56 *bHLH* genes, respectively, originated from segmental duplication events. These duplication events provide a genetic reservoir for functional diversification, where some genes retain ancestral functions while others undergo neofunctionalization or subfunctionalization due to sequence variation and divergence in expression patterns. Furthermore, bHLH proteins often interact with other transcription factors, such as MYB and WD40 proteins, to form multi-protein complexes (e.g., the MBW complex) that coordinately regulate the biosynthesis of secondary metabolites, including anthocyanins and floral volatiles [11]. This interaction enhances their regulatory potential in plant adaptation and trait development [12,13].

*Rosa roxburghii* Tratt., commonly known as Cili or chestnut rose, is a perennial shrub of the Rosaceae family valued for its nutritional and medicinal fruit. The fruit is particularly rich in vitamin C and often referred to as the ‘King of Vitamin C’, and also contains other bioactive compounds like polyphenols and flavonoids [14,15]. Recent advancements, notably the release of the *R. roxburghii* genome sequence [16], have accelerated research into the molecular mechanisms underlying its key agronomic traits, including L-ascorbic acid content, fruit development, stress tolerance, and the accumulation of secondary metabolites [17,18,19,20]. Comparative analysis of *bHLH* genes across Rosaceae species indicate that the expansion of the bHLH gene family in this lineage has been driven by both ancient and recent duplication events. Furthermore, these genes have been shown to play significant roles in responses to abiotic stresses such as cold and drought [21,22,23]. Despite these advancements, a systematic investigation of the bHLH transcription factor family in *R. roxburghii* remains limited, resulting in a knowledge gap in our understanding of the genetic regulation underlying its economically important traits.

To address this research gap, we conducted a systematic genome-wide analysis of the bHLH transcription factor family in *R. roxburghii*. The specific objectives of this study were as follows: (1) identify and characterize all putative *bHLH* genes along with their physicochemical properties; (2) examine their phylogenetic relationships, gene structures, conserved motifs, and chromosomal distributions; (3) investigate gene duplication events and evolutionary constraints; (4) analyze their expression profiles in developing fruits. Our results provide the first comprehensive genomic characterization of the *RrbHLH* gene family, offering insights into the evolutionary dynamics underlying its functional diversification. This work establishes a solid foundation for future functional genomic studies and facilitates molecular breeding strategies for the genetic improvement of this economically important fruit crop.

## 2. Results

### 2.1. Identification and Physicochemical Characterization of the RrbHLH Gene Family

**Figure 1:**
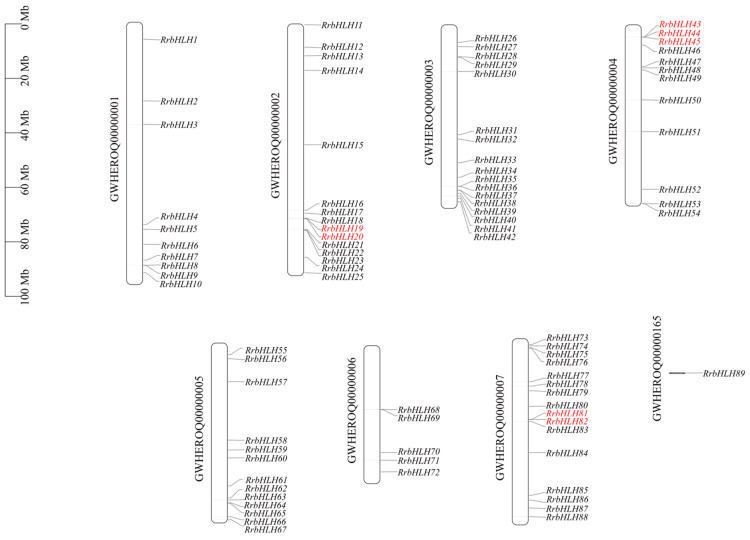
Schematic depiction of the chromosomal position of *RrbHLH* genes. At the left of each chromosome is the corresponding chromosome number. Tandem duplicates are indicated with red letters.

A genome-wide identification of *bHLH* members in *R. roxburghii* was conducted using integrated BLAST and HMMER searches using TBtools (v2.371), followed by validation of the conserved bHLH domain via the Pfam and SMART database. This comprehensive analysis identified 89 non-redundant bHLH transcription factors within the *R. roxburghii* genome. Chromosomal mapping revealed that 88 *RrbHLH* genes were distributed across chromosomes 1 to 7, with gene counts per chromosome ranging from 5 to 17 (Figure 1; Table S1). These genes were systematically designated as *RrbHLH1* to *RrbHLH88* based on their chromosomal positions, while the remaining gene (*RrbHLH89*) was located on a genomic scaffold.

Subsequent analyses of the physicochemical properties of RrbHLH proteins revealed substantial variation among family members. The lengths of these proteins ranged from 142 amino acids (RrbHLH9) to 912 amino acids (RrbHLH14), with corresponding molecular weights varying from approximately 16.39 kDa to 100.19 kDa (Table S1). Theoretical isoelectric points (pI) ranged from 4.71 to 10.32, indicating diverse charge characteristics at physiological pH (Table S1). Analysis of the instability index suggested that the majority of RrbHLHs exhibited values exceeding 40, thus classifying them as unstable. Predictions of subcellular localization indicated that 83 RrbHLHs are localizated in the nucleus, consistent with their anticipated function as transcription factors. The remaining six proteins demonstrated alternative localizations: two each in cytosol–nucleus dual localization (RrbHLH9, RrbHLH60), cytosol-only (RrbHLH34, RrbHLH44), and chloroplast (RrbHLH17, RrbHLH35). Collectively, these findings reveal considerable diversity in the physicochemical properties of the *RrbHLH* family members.

### 2.2. Phylogenetic Analysis of RrbHLHs

**Figure 2:**
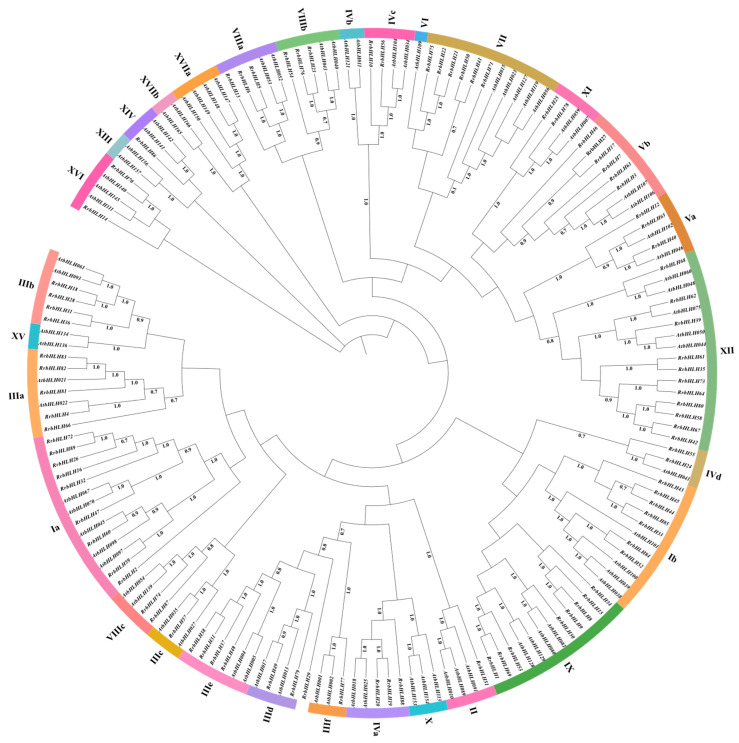
Phylogenetic tree analysis of *AtbHLHs* and *RrbHLHs*. The neighbor-joining (NJ) tree was generated using MEGA12 based on the full-length bHLH protein sequences. The numerals around the phylogenetic tree circle represent the subfamily name of *bHLH* genes. The numbers on the branches indicate the support rates.

**Figure 3:**
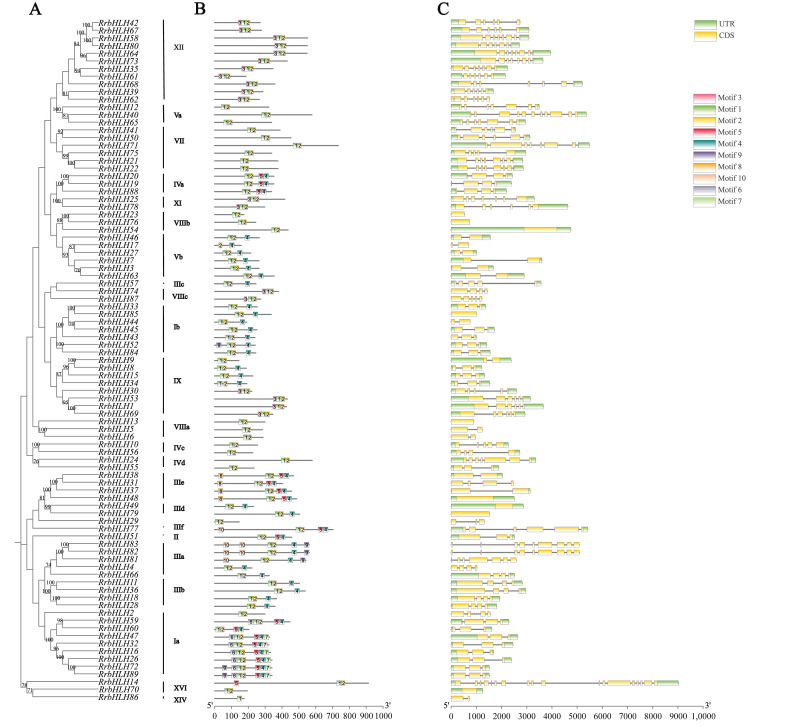
Phylogenetic tree, protein-conserved motif, and intron-exon organization of *RrbHLH* gene family. (**A**) Phylogenetic tree relationship of *RrbHLHs* (**B**) Conserved motif analysis of *RrbHLHs* (**C**) exon intron organization analysis of *RrbHLHs*. Note: Motif analysis was carried out by MEME suite, and the distinct 10 conserved motifs were marked with different numbers. Yellow and green boxes indicate the exon and intron, respectively.

To elucidate the evolutionary relationships among *RrbHLH* genes, we constructed a phylogenetic tree utilizing full-length protein sequences from 89 *R. roxburghii* and 76 *AtbHLH* genes. The *bHLH* genes were classified into 17 families, comprising a total of 30 subfamilies (Figure 2). These *RrbHLH* members were distributed across 23 subfamilies, while 7 subfamilies (IVb, VI, X, XIII, XV, XVIIa, and XVIIb) lacked representatives of *RrbHLH* genes. Subfamily XII contained the highest number of *RrbHLH* members, followed by subfamilies Ia and IX (Figure 2 and Figure 3A). In contrast, subfamilies II, IIIc, IIIf, and XIV each contained only a single *RrbHLH* member. Additionally, *RrbHLH29* was not assigned to any established subfamily and was therefore designated as an orphan gene (Figure 2 and Figure 3A).

To explore the potential functional diversification of *RrbHLHs* from an evolutionary perspective, we mapped the distribution of functionally annotated *bHLH* orthologs (defined by NCBI CDD, e.g., bHLH_AtPIF_like, bHLH_AtAMS_like) onto our phylogenetic subfamilies (Figure S1). This analysis revealed distinct evolutionary patterns linked to functional specialization. *RrbHLH* orthologs of functionally niche types, such as bHLH_AtPIF_like, were exclusively clustered within specific subfamilies (e.g., Subfamily VII), suggesting their evolutionary trajectory towards specialized environmental sensing, like light perception. Conversely, orthologs of proteins with bHLH_AtbHLH_like were found in distinct lineages (Subfamilies VIIIa and IX). Furthermore, functions related to stress response and specialized metabolism (e.g., bHLH_AtAIB_like, bHLH_AtMYC1_like) primarily arose through internal diversification within large, ancient subfamilies. Most notably, Family III harbors diverse roles ranging from hormone signaling (Subfamily IIId) to anthocyanin biosynthesis (Subfamily IIIf). Collectively, the functional landscape of RrbHLH proteins appears to be shaped by three core evolutionary strategies: lineage specialization, convergent recruitment, and internal radiation.

### 2.3. Motif Composition and Gene Structure of the RrbHLH Family

The conserved motif analysis of the 89 *RrbHLH* protein sequences revealed a total of 10 distinct motifs, designated as motif1 through motif10. The detailed information of these motifs was presented in Figure S2. Notably, these motifs with varying compositions among the different protein members (Figure 3B). Motif1 and motif2, which form the core bHLH domain, were found adjacent to each other in the sequence. Six subfamilies—Va, VII, VIIIa, VIIIb, IVc, and IVd—contained only these two motifs, with the exceptions of *RrbHLH17*, which possesses only motif2, and *RrbHLH86*, which possesses only motif1. Most subfamilies exhibited characteristic motif patterns; for example, all 11 members of subfamily XII contained motifs 1, 2, and 3, while subfamily IIIb contained motifs 1, 2, and 4 (Leucine Zipper). Variations were also noted within certain subfamilies: although most members of subfamily Ib contained motifs 1, 2, and 4, *RrbHLH52* additionally included motif 9 (Zinc Finger). A similar pattern was observed in subfamily Vb. Furthermore, in subfamily IX, some members contained motifs 1, 2, and 4, while others contained motifs 1, 2, and 3.

Gene structure analysis revealed that the lengths of *RrbHLH* genes ranged from 522 bp to 9024 bp, with varying numbers of exons (Table S1). *RrbHLH14* exhibited the highest number of exons (18), while nine genes were found to be intronless (Figure 3C). Most members within the same subfamily displayed similar exon–intron organizations. For example, all members of subfamilies Vb, VIIIb, and IVa consistently contained 2, 1, and 4 exons, respectively. Additionally, most members of subfamilies Ia and Ib shared similar structural patterns. In contrast, members of other subfamilies showed considerable structural divergence (Figure 3C). Collectively, these results demonstrate the complex gene structure of the *RrbHLH* family.

### 2.4. Characterization of Key Amino Acid Residues in Conserved bHLH Region

**Figure 4:**
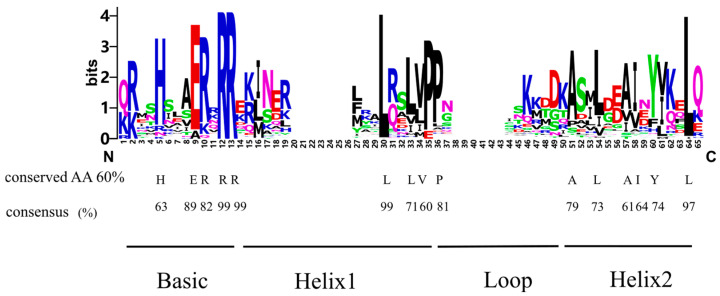
Multiple sequence alignment of *RrbHLHs* and proportion of conserved amino acids. The label “conserved AA 60%” denotes that only amino acid sites with a conservation level exceeding 60% are displayed, along with their specific consensus values (shown as percentages).

To predict DNA-binding properties, we aligned the amino acid sequences of the bHLH domain of RrbHLH proteins. The results show that the first 13 amino acids correspond to the basic region. Among RrbHLH proteins, 89% contain a glutamic acid (E) residue at position 9, which directly interacts with the CA nucleotides of the hexanucleotide E-box sequence (Figure 4). Furthermore, more than 60% of the proteins exhibit a histidine (H) at position 5 and an arginine (R) at position 13, forming the characteristic configuration H5-E9-R13 that interacts with the canonical hexanucleotide E-box motif (5′-CANNTG-3′). Other frequently occurring basic residues, such as arginine at positions 10 and 12, are also highly conserved in RrbHLH proteins, with consensus rates of 82% and 99%, respectively (Figure 4).

The HLH region facilitates the formation of homodimeric or heterodimeric complexes between bHLH proteins. Our analysis indicated that the hydrophobic residues Leu-30 in helix 1 and Leu-64 in helix 2 are the most conserved across the *RrbHLH* family (99% and 97%, respectively) (Figure 4). Additionally, a conserved proline (P) interrupts the first helix and initiates a loop of variable length, typically consisting of six to eight residues.

### 2.5. Cis-Acting Element Analysis in Promoter of RrbHLHs

**Figure 5:**
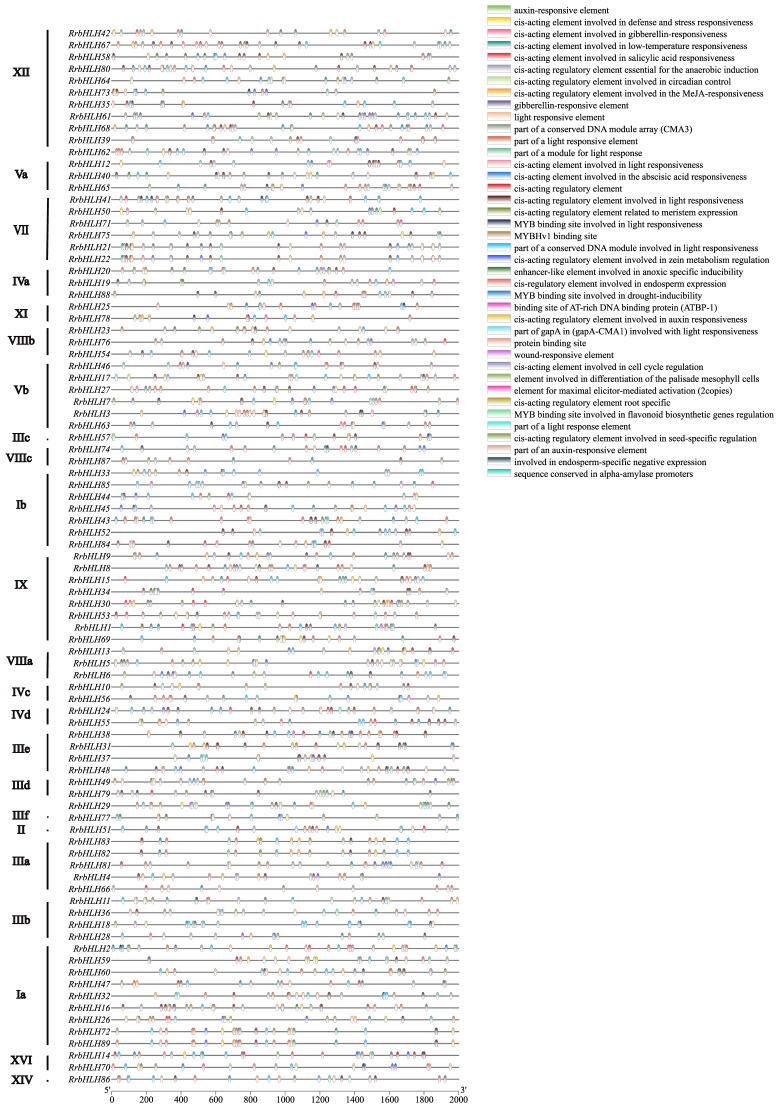
The cis-acting elements detected in the promoter regions of *RrbHLH* genes. The distributions of cis-acting elements in the 2000 bp upstream promoter are shown. The different functions of cis-acting elements are represented by different colors, as shown on the right.

To investigate the transcriptional regulation of *RrbHLH* genes, we analyzed the 2000 bp upstream promoter sequences of all 89 *bHLH* members, identifying a total of 2435 *cis*-acting elements. Each *RrbHLH* gene exhibited multiple types of *cis*-acting elements, with light-responsive elements found in all members, indicating a conserved potential for light-mediated regulation across the family (Figure 5). These elements were subsequently classified into five functional categories.

Hormone-responsive elements, including ABRE, TCA-element, TGACG-motif, and CGTCA-motif, are associated with responses to the abscisic acid, salicylic acid, methyl jasmonate, and other phytohormones, suggesting their roles in hormonal signaling during growth and development. Stress-responsive elements such as LTR, MBS, and TC-rich repeats are implicated in adaptation to low temperature, drought, and defense responses. Tissue-specific elements, including GCN4_motif and HD-Zip 1, may regulate expression in specific organs or cell types, such as endosperm, root, meristem, and mesophyll tissues. Metabolism-related elements, including MBSI and O2-site, are potentially involved in the regulation of flavonoid biosynthesis and zein metabolism regulation. Additionally, core promoter elements such as the CAAT-box and enhancer regions, along with transcription factor-binding sites like those for MYB, were identified as fundamental components of the transcriptional machinery. This systematic profiling reveals a complex regulatory architecture of *RrbHLH* genes and provides insights into their potential functions in plant development, stress adaptation, and metabolic regulation.

### 2.6. Expression Profiling of the RrbHLH Gene Family in Rosa roxburghii

To further understand the functional diversity of *RrbHLH* genes, we analyzed their expression patterns during fruit development. Initially, we compared the expression levels between GA_3_-treated and untreated fruits at 120 days post-pollination. Hierarchical clustering analysis categorized the 89 *RrbHLH* genes into three distinct clusters based on their expression profiles (Figure S3). Approximately half of the *RrbHLH* genes (41 genes) exhibited low expression levels, including *RrbHLH16*, *RrbHLH60*, *RrbHLH19*, and *RrbHLH85*. In contrast, 23 genes, such as *RrbHLH42* and *RrbHLH78*, demonstrated consistently high expression. The remaining genes displayed moderate expression levels.

A total of 30 *RrbHLH* genes exhibited significant differential expression in fruits following GA_3_ treatment (Figure S3). These genes were categorized into 16 subfamilies, including four from subfamily XII, and three each from subfamilies Ia, IIIb, IIIe, and IX (Figure 1 and Figure S3). Notably, four *RrbHLH* genes from different subfamilies—*RrbHLH57* (IIIc), *RrbHLH24* (IVd), *RrbHLH55* (IVd), and *RrbHLH34* (IX)—demonstrated increased expression levels.

**Figure 6:**
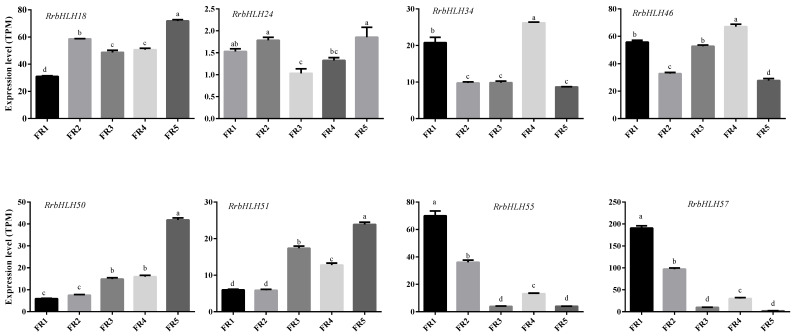
Expression patterns of *RrbHLH* genes across different fruit development stages in *Rosa roxburghii* based on the analysis of transcriptome data. Different letters above the bars represent significant differences at *p* < 0.05 level.

We further examined the expression patterns of *RrbHLH* genes across various stages of fruit development. A total of 75 *RrbHLH* genes exhibited dynamic expression changes during this process, which allowed us to categorize them into two distinct clusters (Figure S4). Notably, we concentrated on genes that showed significant expression differences following GA_3_ treatment (Figure S3). For instance, *RrbHLH55* and *RrbHLH57* were relatively highly expressed during the early developmental stage FR1, while *RrbHLH34* and *RrbHLH46* showed elevated expression levels in FR4. By contrast, *RrbHLH18*, *RrbHLH24*, *RrbHLH50*, and *RrbHLH51* showed higher expression in FR5 (Figure 6). These results suggest that these genes may have distinct roles at different stages of fruit development.

### 2.7. Gene Duplication Events and Functional Divergence of RrbHLHs

**Figure 7:**
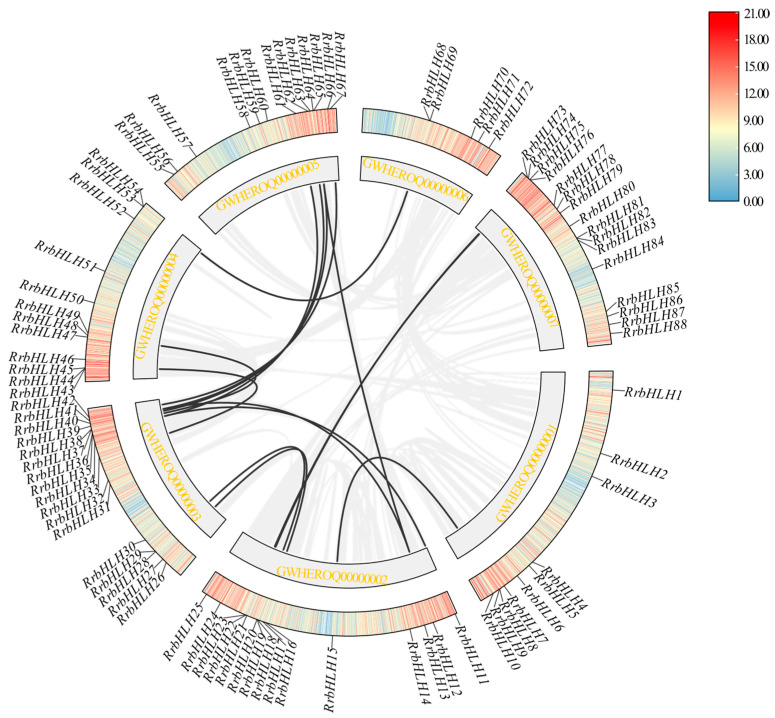
Circos-plot showing the collinearity of the *RrbHLH* genes.

To elucidate the expansion mechanism of the *RrbHLH* gene family, we analyzed gene duplication events using MCScanX. A total of 36 *RrbHLH* genes were identified as products of duplication events, forming 21 duplicated pairs. They included 4 tandem duplication pairs primarily located on chromosomes 2, 4, and 7 (Figure 1), as well as 17 segmental duplication pairs involving 29 genes distributed across all seven chromosomes (Figure 7). All duplicated pairs clustered within the same subfamily (Table S2; Figure 2); for instance, *RrbHLH19*/*20* belongs to subfamily IVa, while *RrbHLH81*/*82* is part of subfamily IIIa. Notably, *RrbHLH*44 formed separately duplication pairs with *RrbHLH*43 and *RrbHLH45*. One segmental pair, *RrbHLH72*/*89*, included a gene located on a scaffold (Figure 1). These findings suggest that segmental duplication served as the primary driver of expansion in the *RrbHLH* family. Selection pressure analysis revealed that all calculable duplicated pairs exhibited non-synonymous (Ka) and synonymous (Ks) substitutions rates below 1, indicating the action of purifying selection during evolution (Table S2). The Ka/Ks ratio for the segmental pair *RrbHLH37*/*48* could not be determined due to high sequence divergence. Divergence times of the duplicated pairs, estimated from Ks values, ranged from approximately 41.07 to 121.23 million years ago.

**Figure 8:**
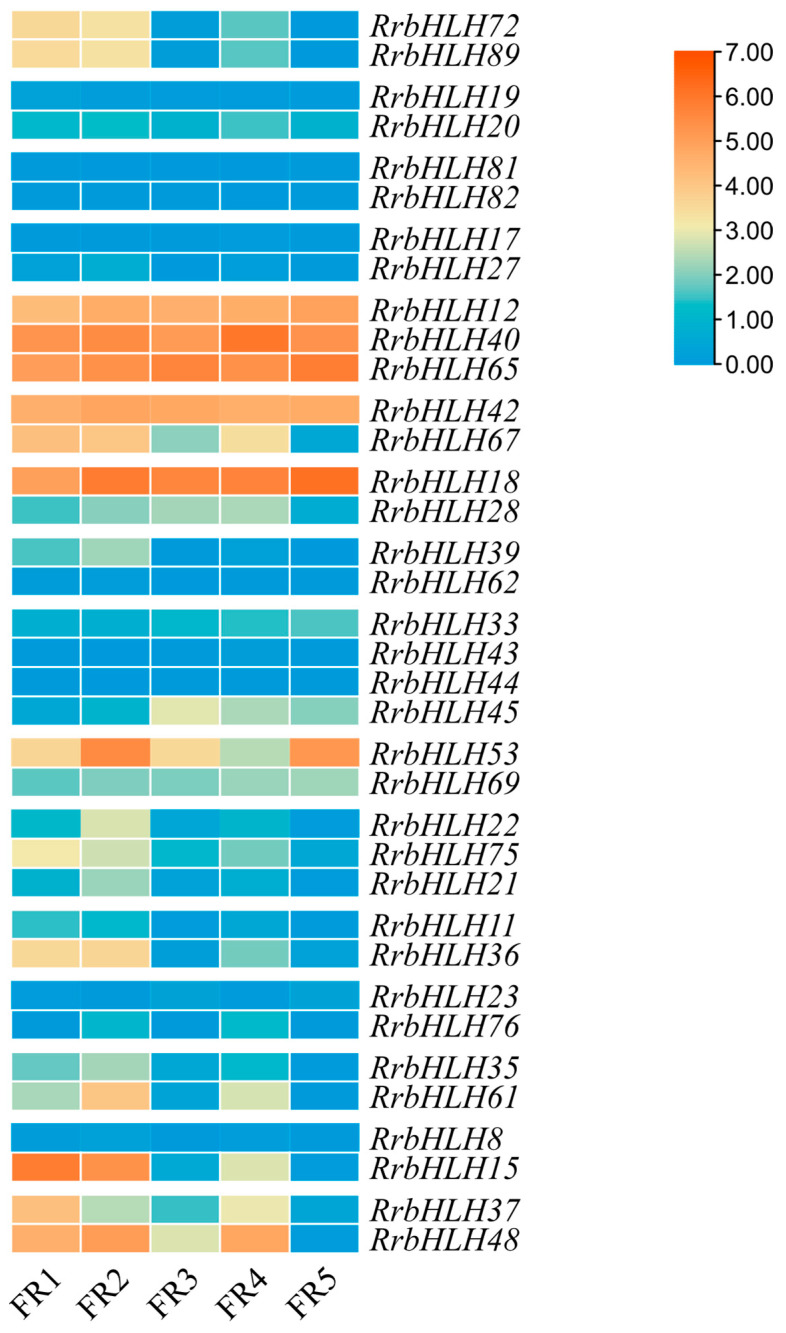
The expression patterns of the duplicated *RrbHLH* pairs across various stages of fruit development.

We analyzed the expression patterns of duplicated gene pairs across various stages of fruit development. The results showed that the two tandem duplication pairs (*RrbHLH43*/*RrbHLH44* and *RrbHLH81*/*RrbHLH82*) exhibited no detectable expression. In contrast, one segmental duplication pair (*RrbHLH72*/*RrbHLH89*) displayed similar expression profiles (Figure 8). However, most segmental duplication pairs demonstrated divergent expression patterns during fruit development. For example, *RrbHLH42* maintained relatively high expression across all stages, while its duplicated counterpart *RrbHLH67* exhibited a decreasing trend during the first three stages, followed by an increase at the fourth stage and a subsequent decline at the fifth stage. These findings suggest that duplications have significantly contributed to the functional diversification within the *RrbHLH* family.

### 2.8. Co-Expression Networks of Duplicated RrbHLH Pairs

To further investigate the expression regulatory patterns of duplicated *RrbHLH* pairs, we constructed a weighted gene co-expression network (WGCNA). A total of 22,544 genes were grouped into 33 distinct modules with 58 *RrbHLHs* distributed across 16 modules (Figure S5). Among these, 22 duplicated *RrbHLHs* were allocated to 13 modules, with most gene pairs assigned to different modules (Table S2), suggesting divergence in their biological functions. For instance, *RrbHLH18* and *RrbHLH28* were located in the midnight blue and light yellow modules, respectively (Table S2). Notably, this duplicated pair also showed differential expression levels across various fruit developmental stages (Figure 8).

**Figure 9:**
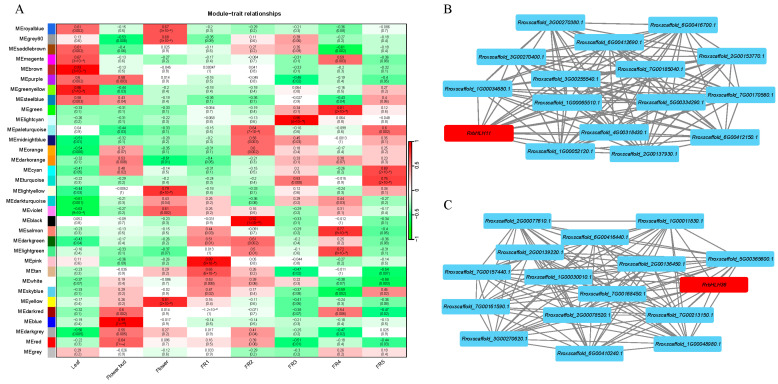
The heatmap shows the module-trait correlation (**A**). Each row represents a gene co-expression module, and each column represents a specific tissue. The number in each cell indicates the correlation coefficient, with the corresponding p-value in parentheses. Red and green colors denote positive and negative correlations, respectively. Weighted co-expression gene network centered on *RrbHLH11* (**B**) and *RrbHLH36* (**C**).

We analyzed the correlation between the 33 modules and eight tissue types (Figure 9A). The results revealed that duplicated *RrbHLHs* present in the 13 modules were correlated with all tissues except FR3. For example, *RrbHLH72* and *RrbHLH8*9 were clustered in the red module and were primarily associated with flower bud (Table S2 and Figure 9A). Meanwhile, *RrbHLH11* and *RrbHLH36* were assigned to the green and purple modules, respectively, and both separately showed correlation with FR4 and leaf (Table S2 and Figure 9B,C). Analysis of co-expressed genes further indicated that the *RrbHLH11*/*36* duplicated pair participates in distinct gene co-expression networks (Figure 9B,C). Together, these findings suggest that gene duplication has contributed to functional divergence within the *RrbHLH* family.

### 2.9. Exon–Intron Structural Divergence Among Duplicated RrbHLH Pairs

**Figure 10:**
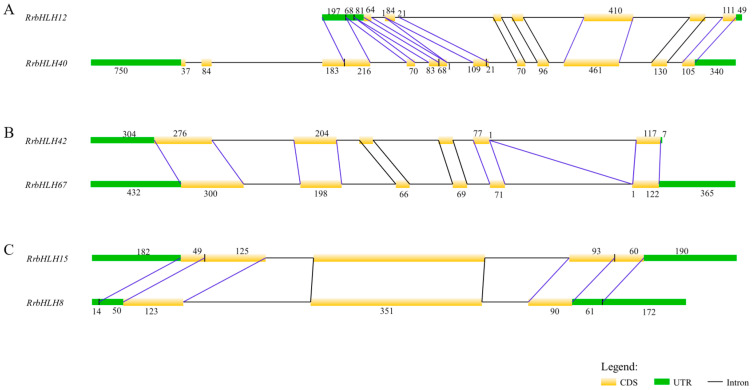
The exon–intron structures of the representative duplicated pairs and three scenarios of structural divergence: (**A**) insertion/deletion, (**B**) dissolution/joining of exons, and (**C**) exonization/pseudoexonization. Note: The numbers indicate the exon length for each pair. A blue line connects pairs that differ in exon length, in contrast to a black line, which indicates pairs of identical length.

Structural analysis revealed a significant divergence in the exon–intron organization among the 21 duplicated *RrbHLH* gene pairs. Of these, 7 pairs (33.3%) exhibited differences in exon numbers, indicative of major structural reorganization, while the remaining 14 pairs (66.7%) displayed length variations in one or more homologous exons despite maintaining identical exon counts (Figure S6). Further investigation through precise sequence alignment identified three fundamental mechanisms driving these structural variations. First, intraexonic indels were universally observed across all duplicated pairs, manifesting as small insertions or deletions within exons that directly contribute to exon length divergence; complete exon indels were particularly rare and detected only in pairs involving *RrbHLH40*/*12*, *RrbHLH40*/*65*, and *RrbHLH81*/*82* (Figure 10). Second, the widespread phenomenon of exon dissolution and joining occurred in 20 of the 21 pairs, primarily involving either the splitting of single exons into multiple smaller exons or the merging of multiple exons into larger units. An example of this is the alignment of the fifth exon of *RrbHLH42* with both the fifth and sixth exons of *RrbHLH67* (Figure 10). Third, the processes of exonization and pseudoexonization facilitated the interconversion between exonic and non-exonic sequences, with a particularly illustrative example being the alignment of terminal exon regions in *RrbHLH15* with untranslated regions in *RrbHLH8* (Figure 10). Collectively, these structural divergences, particularly those affecting exon composition and length, establish a plausible molecular basis for the functional differentiation observed among duplicated *RrbHLH* gene pairs.

## 3. Discussion

The bHLH transcription factor family is one of the largest and most functionally diverse gene families in plants, playing pivotal roles in regulating growth, development, stress responses, and secondary metabolism [8,24]. Recent advancements in high-throughput sequencing have enabled the genome-wide characterization of gene families in non-model species. Utilizing the recently released genome of *R. roxburghii*, this study identified 89 *RrbHLHs* in this nutritionally valuable fruit crop.

Sequence analysis of RrbHLH proteins confirmed a high degree of conservation at functionally critical positions within the bHLH domain. Key residues in the basic region—including H5, E9, and R13, which are involved in direct DNA contact—were broadly conserved, as were hydrophobic leucine residues in the HLH region that stabilize the dimerization interface [24,25,26]. This conservation indicates that the core DNA-binding and dimerization functions of the ancestral bHLH domain have been maintained in *R. roxburghii*. Beyond this conserved core, several *RrbHLH* members contain additional functional motifs, such as leucine zippers, which may fine-tune dimerization specificity, transcriptional activity, or protein stability. Phylogenetic analysis revealed that these accessory domains are restricted to particular subfamilies (e.g., Ia, IIIa and IIIe), consistent with independent acquisition events during evolution. This pattern supports the occurrence of domain shuffling in the expansion of the bHLH family, as reported in broader studies of plant bHLH proteins [24,26]. The modular addition of such domains likely provided a structural foundation for functional diversification, allowing RrbHLH proteins to participate in more specialized regulatory networks during plant development and stress responses.

The bHLH transcription factor family has undergone significant expansion during plant evolution [24]. In *R. roxburghii*, we identified 89 *bHLH* genes, a number comparable to some diploid Rosaceae species but smaller than in *Arabidopsis* [7] or rice [22], which may be attributed to variations in genome size, ploidy, and lineage-specific duplication events. Gene duplication has been a primary driver the expansion of the *bHLH* family [27,28]. In *R. roxburghii*, we detected 21 duplicated gene pairs, comprising 17 segmental and 4 tandem duplication pairs. This pattern aligns with the expansion mechanisms observed in other Rosaceae species [23]. Comparative analysis across five Rosaceae species revealed that WGD and segmental duplication played critical roles in pear and apple, whereas dispersed duplication was more prominent in peach, strawberry, and Chinese plum [23]. The estimated divergence times of the *RrbHLH* duplication pairs, based on Ks values, span a wide range (41~121 MYA), reflecting multiple rounds of duplication throughout evolutionary history. Our findings align with broader trends observed in Rosaceae bHLH research. For example, studies in pear identified 198 *PbbHLH* genes, with expansion driven largely by recent WGD events (30~45 MYA), while peach, strawberry, and Chinese plum experienced more ancient duplication events [23]. Notably, seven *Arabidopsis* subfamilies (IVb, VI, X, XIII, XV, XVIIa, XVIIb) are absent in *R. roxburghii*, possibly due to lineage-specific gene loss or functional consolidation.

After gene duplication, copies can acquire novel functions or undergo division of labor through sequence variation, expression divergence, and structural reorganization. In this study, most duplicated *RrbHLH* pairs exhibited Ka/Ks ratios of less than 1, indicating the action of purifying selection. Similar patterns were observed in the bHLH families of the Rosaceae, where most gene pairs evolved under negative selection [23]. Several *RrbHLH* segmental pairs displayed distinct expression profiles across various fruit developmental stages, implying subfunctionalization or neofunctionalization. Structural analysis further revealed three main types of post-duplication variation: (i) intraexonic insertions/deletions, (ii) exon dissolution/joining, and (iii) exonization/pseudoexonization. These structural alterations could influence mRNA splicing, stability, or protein domain architecture [29], thereby providing a molecular basis for functional differentiation between duplicates.

Members of the same bHLH subfamily are frequently involved in analogous biological processes. Most bHLH proteins in *Arabidopsis* have been functionally characterized [7,30] and the functions of *RrbHLHs* will be discussed herein in the context of the corresponding genes from *Arabidopsis.* The best-described bHLH proteins are members of subgroup IIIf, which were involved in the MBW complexes to regulate flavonoid biosynthesis, the differentiation of trichomes, root hair cells and the biosynthesis of seed coat mucilage [7] (Table S3). Therefore, *RrbHLH77* from subfamily IIIf may also be involved in these functions, akin to those observed in *Arabidopsis* (Table S3). A striking exception comes from three *A. thaliana* subfamily Ia proteins, *AtbHLH045*, *AtbHLH097* and *AtbHLH098*, which play nonoverlapping roles in controlling sequential cell fate specification during stomatal differentiation (Table S3). Such apparent functional redundancy is also observed in subfamilies Ib and II, which were involved in iron homeostasis and anther development, respectively. Furthermore, an outstanding example of functional diversification is encountered in subfamily XII, which clusters five *AtbHLHs* and eleven *RrbHLHs* involved in diverse biological roles, including brassinosteroid signaling, phytochrome-dependent photomorphogenic responses (shade avoidance), development of the female reproductive tract, responses to salinity and drought stress, cold stress response and hypocotyl elongation (Table S3). Therefore, future research should prioritize the functional characterization of key *RrbHLH* members, particularly those associated with fruit development, stress adaptation, or metabolite biosynthesis, utilizing genetic and molecular approaches.

## 4. Materials and Methods

### 4.1. Identification of the bHLH Gene Family in Rosa roxburghii

The genome sequence and corresponding annotation files of *R. roxburghii* were retrieved from the China National Center for Bioinformation (https://www.cncb.ac.cn/; accession GWHEROQ00000000). Reference protein sequences of bHLH transcription factors from *Arabidopsis thaliana* and *Oryza sativa* were obtained from the PlantTFDB database (https://planttfdb.gao-lab.org/, accessed on 10 July 2025) to serve as queries. A local BLASTP search was conducted against the complete *R. roxburghii* proteome using TBtools (v2.371) with the bHLH protein sequences from *A. thaliana* and *O. sativa* separately, applying an E-value cutoff of 1 × 10^−5^. The resulting hits from both searches were merged and deduplicated to generate a preliminary candidate set. Concurrently, the Hidden Markov Model (HMM) profile of the bHLH domain (PF00010) was acquired from the Pfam database and used to scan the *R. roxburghii* proteome via the embedded HMMER tool in TBtools (v2.371) [31,32]. Candidate genes identified through both BLAST and HMMER approaches were consolidated into a non-redundant set. The corresponding protein sequences were extracted and further validated using the Pfam (https://www.ebi.ac.uk/interpro/, accessed on 12 July 2025), SMART and NCBI Conserved Domains Database (CDD) to verify the presence of a complete bHLH domain. Any sequences lacking a confirmed bHLH domain were excluded, yielding a final set of high-confidence *RrbHLH* genes.

### 4.2. Analysis of Physicochemical Properties, Conserved Motifs, and Gene Structures

The physicochemical properties of the identified *RrbHLH* proteins, including the number of amino acids (aa), isoelectric point (pI), and molecular weight (MW), were computed using the ‘Protein Parameter Calc’ module in TBtools (v2.371). Conserved motifs within the *RrbHLH* protein sequences were predicted using the ‘Simple MEME Wrapper’ function in TBtools, with the parameters set to a maximum of 10 motifs and an optimum motif width ranging from 6 to 50 amino acids, while all other settings remained at their default values. Gene structure features, specifically the exon–intron organization of each *RrbHLH* gene, were extracted from the *R. roxburghii* genome annotation file in GFF3 format. Visualization of conserved motif distributions and gene structures was subsequently conducted using the ‘Visualize MEME Motif Pattern’ and ‘Visualize Gene Structure’ tools in TBtools, respectively.

### 4.3. Promoter Cis-Acting Element Analysis

The 2000 bp genomic sequences located upstream of the transcription start site of each *RrbHLH* gene were retrieved as putative promoter regions using TBtools (v2.371). These promoter sequences were screened for *cis*-acting elements through the PlantCARE database (https://bioinformatics.psb.ugent.be/webtools/plantcare/html/, accessed on 20 July 2025). The identified *cis*-acting elements were subsequently categorized, and their distributions were visualized using TBtools (v2.371).

### 4.4. Phylogenetic Analysis and Chromosomal Distribution of RrbHLHs

A total of 76 representative *AtbHLH* protein sequences (see Table S3) were retrieved from the TAIR database, covering all known subfamilies [7]. Multiple sequence alignment of the *RrbHLH* proteins and *AtbHLH* representatives was performed using the ClustalW algorithm in MEGA software (v12) with default parameters [33]. A neighbor-joining (NJ) phylogenetic tree was constructed from the aligned sequences using pairwise deletion, the p-distance method, and 1000 bootstrap replicates in MEGA (v12) [33]. The resulting tree was visualized and annotated using the iTOL online platform (https://itol.embl.de/, accessed on 2 August 2025), with the Newick format output from MEGA serving as the input. For chromosomal distribution analysis, the physical locations of the *RrbHLH* genes were mapped onto chromosomes using TBtools (v2.371), based on positional information extracted from the *R. roxburghii* genome annotation file in GFF format.

### 4.5. Gene Duplication and Selection Pressure Analysis

Intra-genomic synteny analysis was conducted to identify duplication events within the *RrbHLH* gene family using the MCScanX algorithm implemented in TBtools (v2.371). Both segmental and tandem duplications were detected, and the syntenic relationships among *RrbHLH* genes were visualized using a Circos plot [34]. For each duplicated gene pair, the rates of non-synonymous (Ka) and synonymous (Ks) substitutions were calculated using the ‘Simple Ka/Ks Calculator’ in TBtools. The Ka/Ks ratio was used to assess selective pressure, with interpretations as follows: Ka/Ks < 1 indicates purifying selection, Ka/Ks ≈ 1 suggests neutral evolution, and Ka/Ks > 1 reflects positive selection. The divergence time (T) for each duplicated pair was estimated using the equation T = Ks/(2 × r), where a substitution rate (r) of 1.5 × 10^−8^ substitutions per site per year was applied for dicot plants.

### 4.6. Expression Profiling Based on Transcriptomic Data

RNA-seq data for *R. roxburghii* were obtained from two publicly available datasets. The first dataset, retrieved from the NCBI SRA database (accession PRJNA1003688), comprised transcriptomes of GA_3_-treated and untreated fruits collected 120 days post-pollination, each with three biological replicates. The second dataset, acquired from the Genome Sequence Archive at the National Genomics Data Center (accession CRA017453), included samples collected across different stages of fruit development. Following quality control, clean reads were aligned to the *R. roxburghii* reference genome using HISAT2 [35]. The expression levels of *RrbHLH* genes were quantified and normalized as transcripts per million (TPM). A heatmap was subsequently generated using TBtools (v2.371) to visualize expression patterns across tissues, and hierarchical clustering was employed to group genes with similar expression profiles.

### 4.7. Weighted Correlation Network Analysis (WGCNA)

The top 50% of transcripts ranked by the expression levels were selected for WGCNA. In this study, we conducted the analysis from 9 samples of leaf, flower and flower bud, except for the abovemetioned 15 different developing fruit tissues. The pickSoftThreshold function was applied to determine an appropriate soft-thresholding power, ensuring that the resulting network followed a scale-free topology. Based on pairwise gene correlations raised to this power, an adjacency matrix was constructed and subsequently converted into a topological overlap matrix (TOM). Module detection was carried out using the dynamic tree cut method, with the following parameters: minModuleSize = 30, mergeCutHeight = 0.15, and deepSplit = 2. The network was configured as “unsigned.”

### 4.8. Structural Divergence Analysis of Duplicated Gene Pairs

Structural divergence among paralogous genes was assessed by comparing their exon-intron organizations in accordance with established methodologies [36]. Gene pairs were classified as structurally divergent if they displayed either differing exon counts or identical exon counts with divergent lengths in at least one homologous exon. To elucidate the mechanisms driving these structural variations, pairwise alignments of genomic sequences were performed using the corresponding cDNA sequences as reference. Through meticulous sequence comparison, three distinct types of structural divergence events were identified: (1) Intraexonic indels, characterized by insertions or deletions within homologous exons; (2) Exon dissolution/joining events, where one exon is split into multiple smaller exons or multiple exons are merged into a single larger exon; and (3) Exonization/pseudoexonization, which involves the evolutionary transition between exonic and intronic sequences among paralogs.

## 5. Conclusions

In this study, we conducted a comprehensive genome-wide analysis of the bHLH transcription factor family in *R roxburghii*, leading to the identification and characterization of 89 non-redundant *RrbHLH* genes. Through phylogenetic analysis with *Arabidopsis thaliana*, these members were classified into 23 subfamilies, with 7 subfamilies (IVb, VI, X, XIII, XV, XVIIa, and XVIIb) notably absent, suggesting lineage-specific evolutionary trajectories. The bHLH members within the same subfamily generally shared similar exon-intron architectures and motif compositions. Subfamily-specific and intra-subfamily variations were observed, highlighting the structural basis for functional diversification. Key DNA-binding and dimerization residues in the *bHLH* domain were highly conserved, preserving the core molecular functions of this domain. Expression profiling revealed dynamic and diverse expression patterns across fruit developmental stages and in response to GA_3_ treatment. Gene duplication analysis identified 21 duplicated pairs, primarily driven by segmental duplication. Although all pairs evolved under purifying selection, their divergent expression patterns and structural variations—including exon indels, dissolution/joining, and exonization/pseudoexonization—provide a mechanistic basis for functional divergence. Collectively, these findings offer fundamental insights into the evolution and potential functions of the *RrbHLH* family.

## Data Availability

The original contributions presented in this study are included in the article/Supplementary Materials.

## Supplementary Materials

The following supporting information can be downloaded at https://www.mdpi.com/article/10.3390/ijms27020912/s1.

## Abbreviations

The following abbreviations are used in this manuscript:

bHLH	basic/helix-loop-helix
WGD	Whole-genome duplication
HMM	Hidden Markov Model
CDD	Conserved Domains Database
MW	Molecular weight
Ka	Non-synonymous substitution
Ks	Synonymous substitution
TPM	Transcripts per million
