# Supplementary figures and images for: Genome-Wide Identification and Evolutionary Analysis of the bHLH Transcription Factor Family in Rosa roxburghii
Source: Int J Mol Sci. 2026 Jan 16;27(2):912. doi: 10.3390/ijms27020912 (PMC12842455; doi:10.3390/ijms27020912)

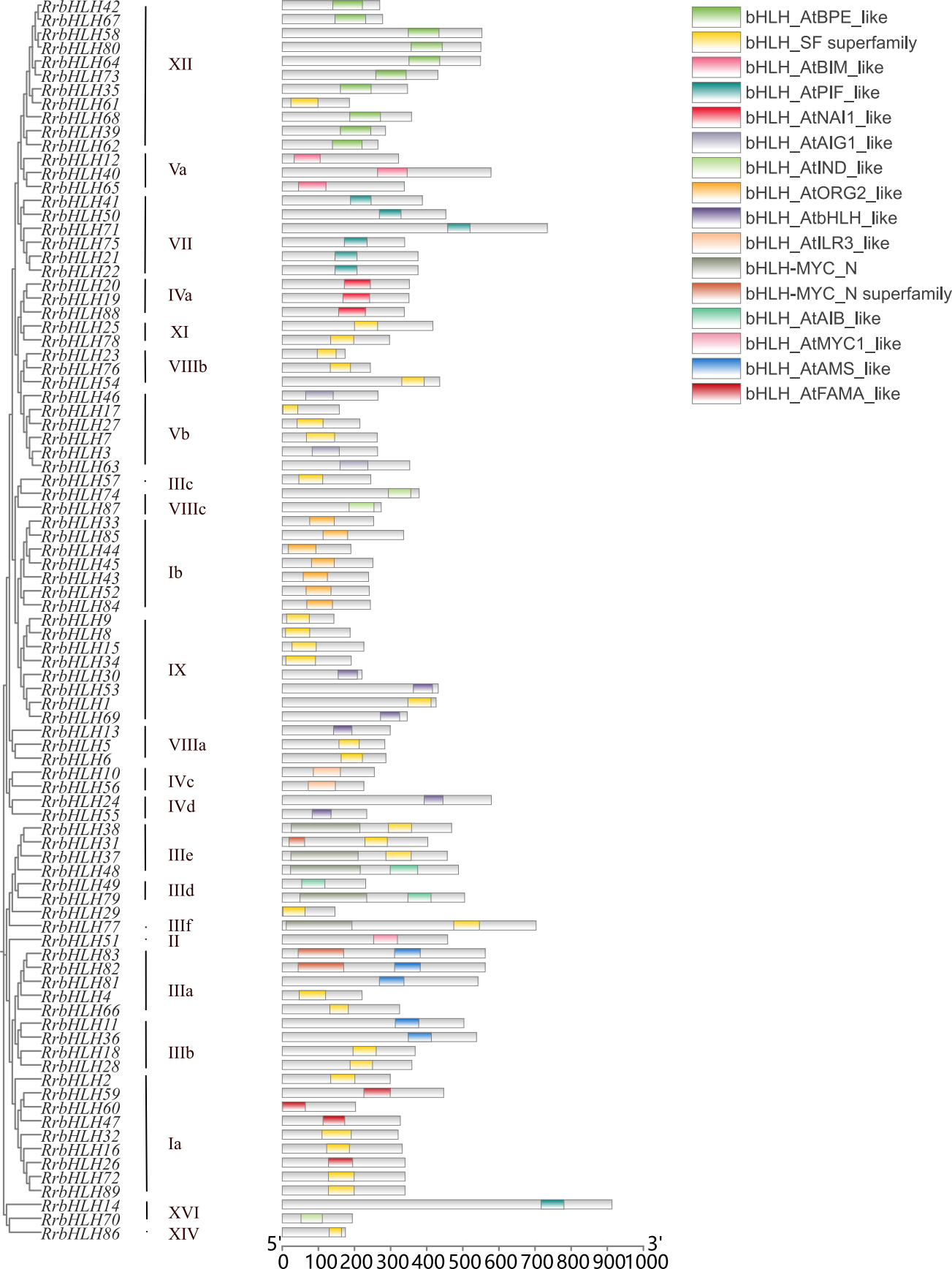

Supplement: Supplementary file 1 [file ijms-27-00912-s001.zip › Figure S1.pdf]

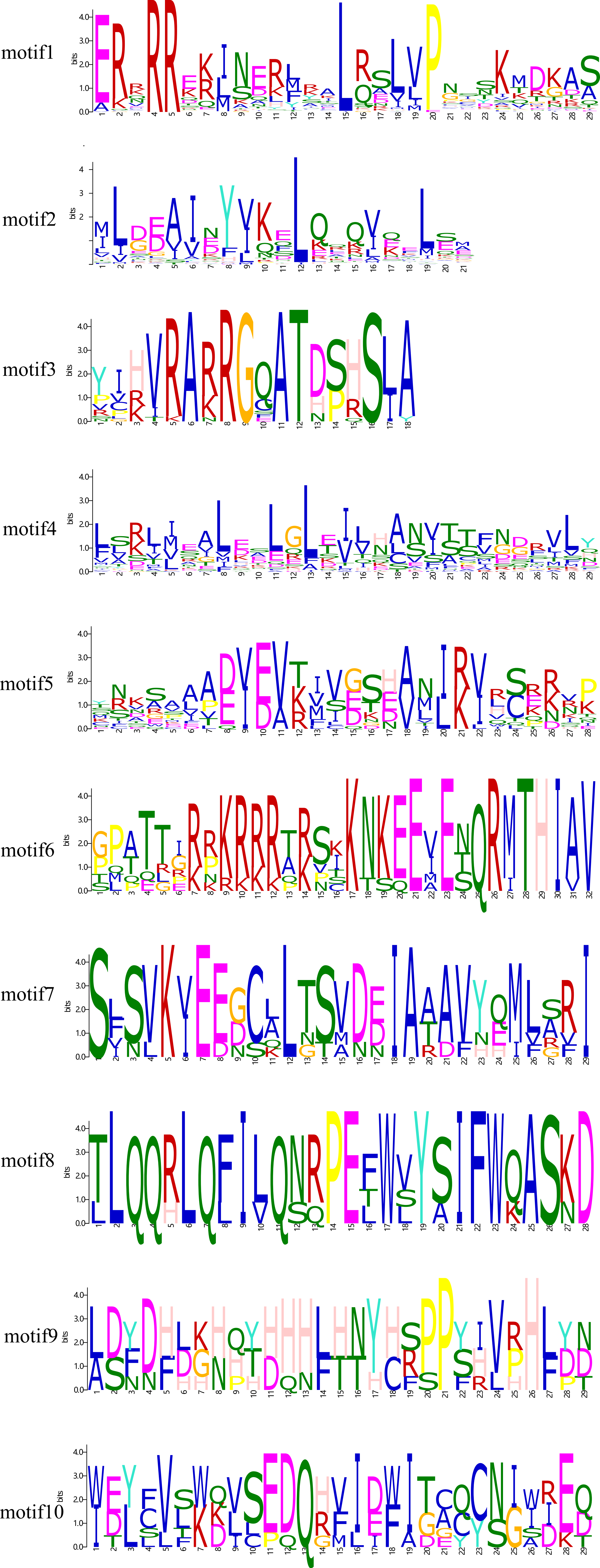

Supplement: Supplementary file 1 [file ijms-27-00912-s001.zip › Figure S2.pdf]

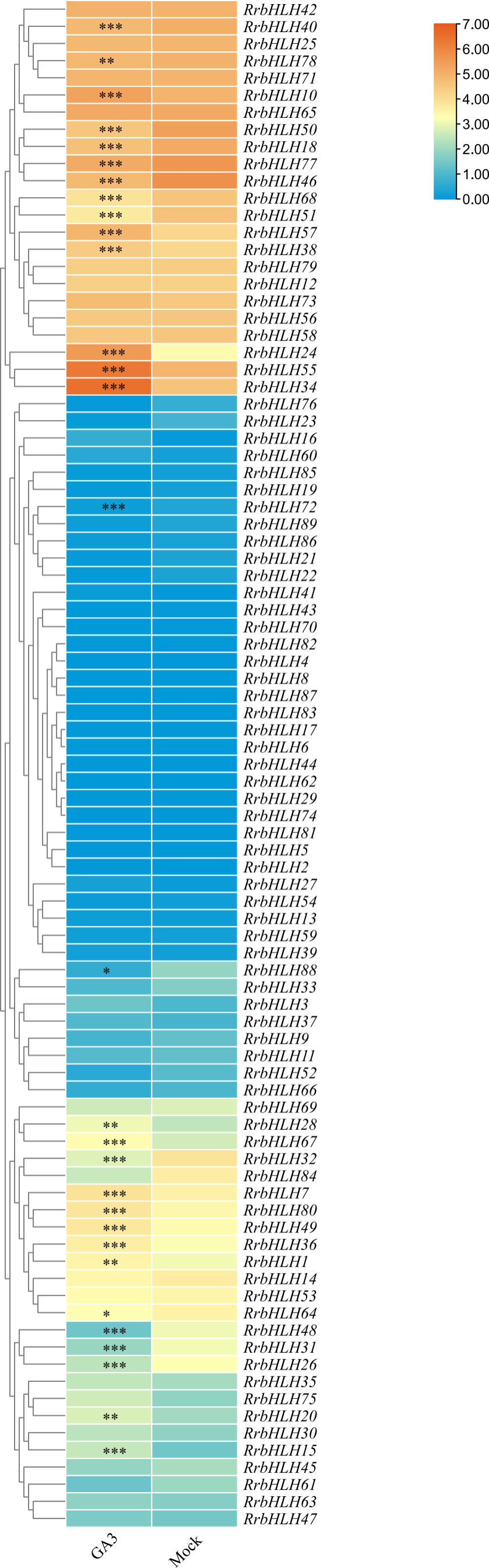

Supplement: Supplementary file 1 [file ijms-27-00912-s001.zip › Figure S3.pdf]

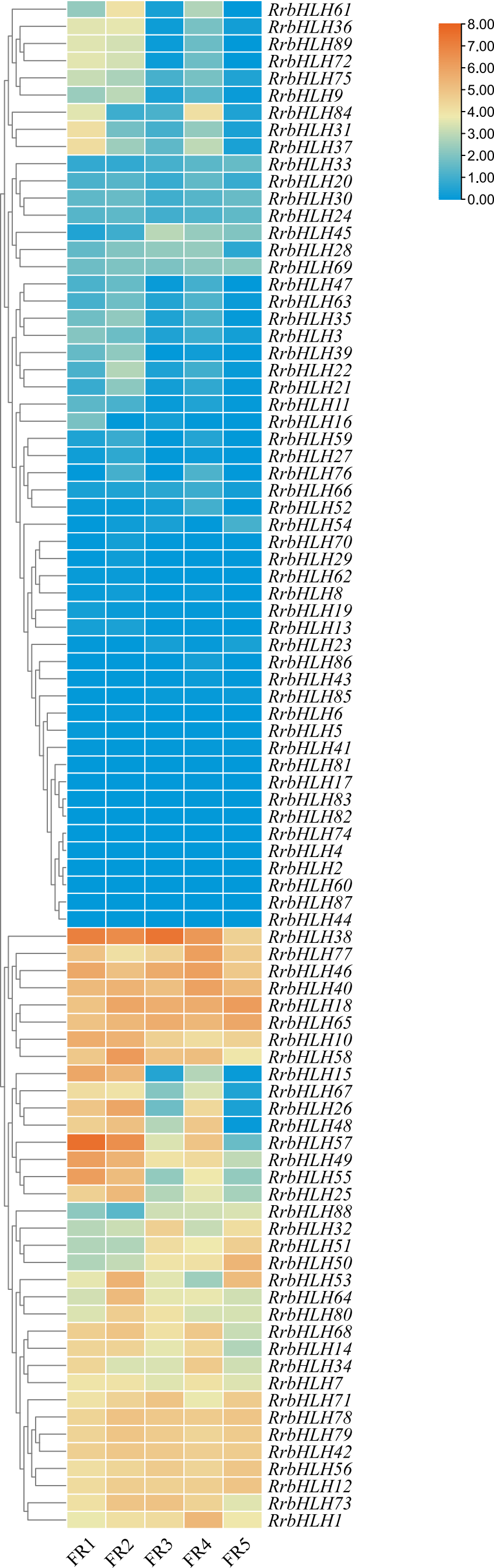

Supplement: Supplementary file 1 [file ijms-27-00912-s001.zip › Figure S4.pdf]

# Cluster Dendrogram

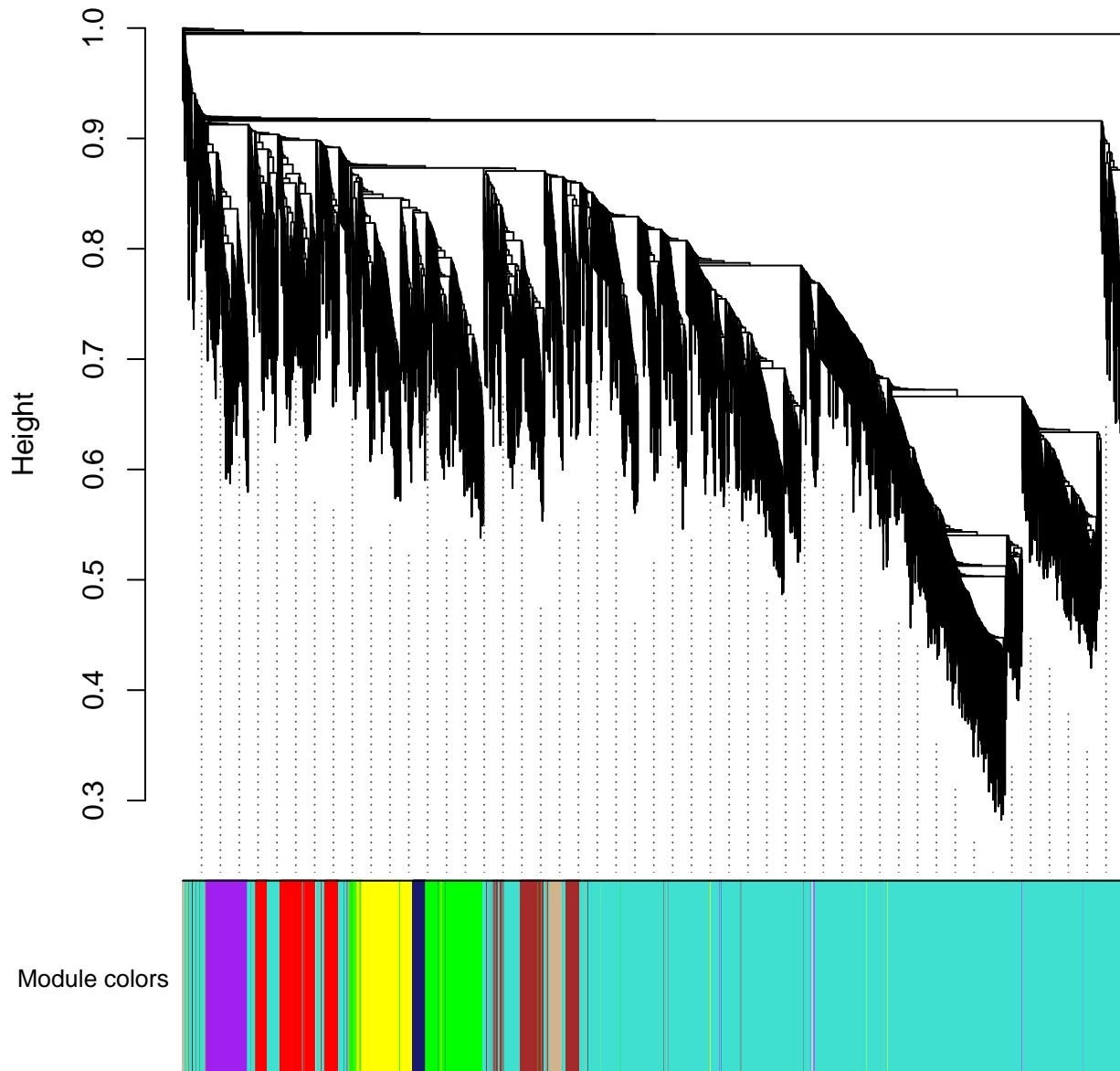

Supplement: Supplementary file 1 [file ijms-27-00912-s001.zip › Figure S5.pdf]
