# Supplementary material for: Genome-Wide Identification and Evolutionary Analysis of the bHLH Transcription Factor Family in Rosa roxburghii
Source: Int J Mol Sci. 2026 Jan 16;27(2):912. doi: 10.3390/ijms27020912 (PMC12842455; doi:10.3390/ijms27020912)
Supplement: Supplementary file 1 [file Figure S6.pdf › Figure S6.pdf]

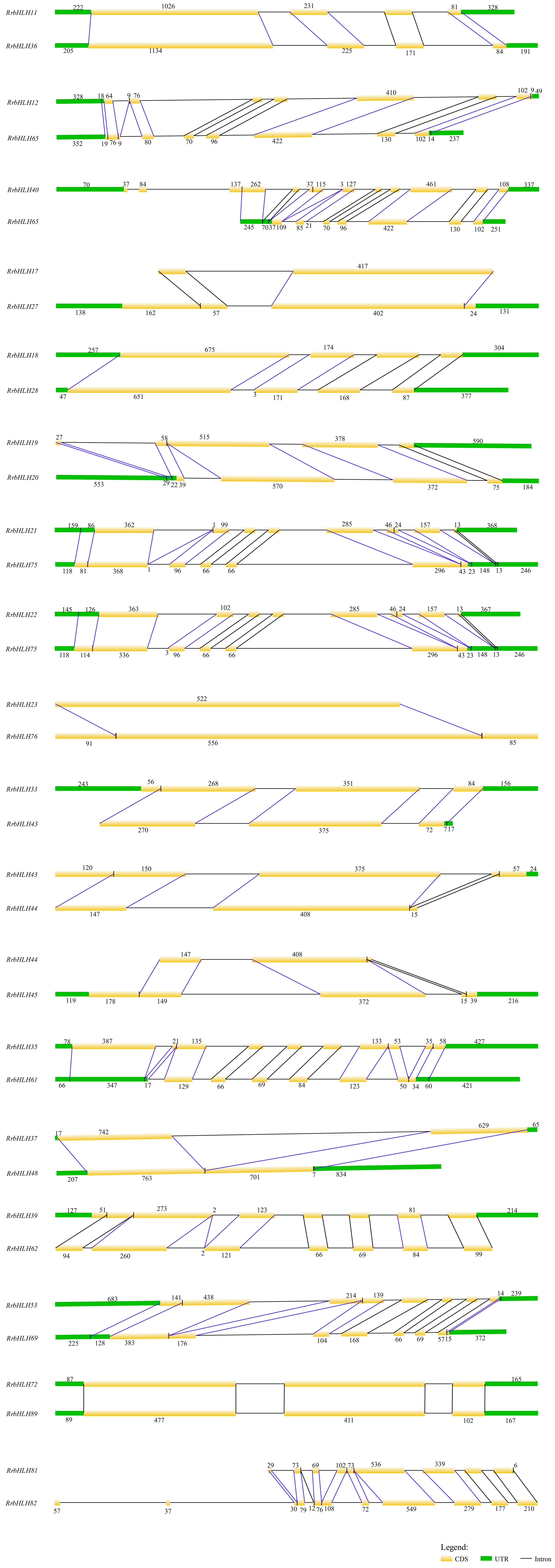

RrbHLH11
RrbHLH36
RrbHLH12
RrbHLH65
RrbHLH40
RrbHLH65
RrbHLH17
RrbHLH27
RrbHLH18
RrbHLH28
RrbHLH19
RrbHLH20
RrbHLH21
RrbHLH75
RrbHLH22
RrbHLH75
RrbHLH23
RrbHLH76
RrbHLH33
RrbHLH43
RrbHLH43
RrbHLH44
RrbHLH44
RrbHLH45
RrbHLH35
RrbHLH61
RrbHLH37
RrbHLH48
RrbHLH39
RrbHLH62
RrbHLH53
RrbHLH69
RrbHLH72
RrbHLH89
RrbHLH81
RrbHLH82
Legend:
CDS
UTR
Intron
